# Supplementary material for: Globally occurring pelagiphage infections create ribosome-deprived cells
Source: Nat Commun. 2024 May 2;15:3715. doi: 10.1038/s41467-024-48172-w (PMC11066056; doi:10.1038/s41467-024-48172-w)
Supplement: Supplementary file 1 — Supplementary Information [file 41467_2024_48172_MOESM1_ESM.pdf]

Supplementary Information to

Globally occurring pelagiphage infections create ribosome-deprived zombie cells

Jan D. Brüwer[1*], Chandni Sidhu[1], Yanlin Zhao[2], Andreas Eich[3], Leonard Rößler[1], Luis H. Orellana[1], Bernhard M. Fuchs[1**]

[1] Max Planck Institute for Marine Microbiology, Bremen, Germany

[2] College of Juncao Science and Ecology, Fujian Agriculture and Forestry University, Fuzhou, China

[3] PSL Research University, EPHE-UPVD-CNRS, UAR 3278 CRIOBE, 98729 Moorea, French Polynesia

Supplementary Material and Methods

Pelagiphage FISH probe design and synthesis

During the Helgoland 2020 spring phytoplankton bloom, 30 PacBio Sequel II metagenomes were sampled between March 3 and May 20 (European Nucleotide Archive (ENA) project PRJEB52999). Sampling, DNA extraction, sequencing, and assembly are described in detail in Sidhu et al.[1]. Briefly, 10 L of unfixed seawater was sampled at the long-term ecological research station Helgoland Roads (54° 11.3′ N, 7° 54.0′ E), filtered sequentially through 10, 3, and 0.2 µm polycarbonate filters (47 mm diameter, Sigma Aldrich, Taufkirchen, Germany) and stored at -80°C until further processing. DNA was extracted from 0.2 µm filters, following Zhou et al.[2]. Samples were sequenced on a PacBio Sequel II (Pacific Biosciences, Menlo Park, CA, USA) using one SMRT cell per sample in long-read HiFi mode at the Max Planck Genome Centre, Cologne, Germany. Raw reads were assembled using Flye (v2.8.3)[3] in *-meta* and *-pacbiohifi* mode.

Potential viruses were identified from assembled contigs using VIBRANT (v1.2.0)[4]. Retrieved sequences were aligned against a database composed of the viral NCBI RefSeq (r203)[5], as well as additional pelagiphage sequences from isolated phages[6-8] using BLASTn (v2.5.0)[9]. Sequences with an alignment to a known phage were further validated by identifying the closest neighbor in a proteomic tree, using VipTree in 2D mode (v1.1.2)[10] with the same database as described above. Retrieved sequences were annotated using DRAM-v.py annotate[11] (with *--use_uniref*) and subsequently aligned with their closest reference using MAFFT (v7.450; algorithm “auto”, scoring matrix “200PAM/k=2”, gap open penalty: 1.53, offset value:0.123)[12] within Geneious (v2022.1.1)[13].

Probes were designed on these alignments with the guideline of 150-300 bp length and minimum 90% nucleotide identity between reference genome and metagenome sequences.

A minimum of 10 probes was designed to target a single phage genome. We aimed to target genes encoding terminases, polymerases, or structural proteins, based on the DRAM-v annotation, where possible. Ambiguous alignment with any other sequence was excluded, using BLASTn against the NCBI webservice (14 February 2023).

Detailed CARD-FISH and direct-geneFISH procedure

Samples were embedded in 0.1% LE agarose (w/v, Biozym, Hessisch Oldendorf, Germany) to minimize cell losses during down-stream handling. Subsequently, cells were permeabilized using 10 mg ml^{-1} lysozyme (Sigma Aldrich, Darmstadt, Germany) in lysozyme buffer (0.05 M EDTA, 0.1 M Tris-HCl, pH 8.0) for 1 hour at 37°C. Next, endogenous peroxidases were inactivated with 0.15% H_2O_2 in methanol for 20 min at room temperature.

For the CARD-FISH hybridization, 0.84 pmol mL^{-1}(final concentration) of 16S FISH probe were added to the hybridization mixture (900 mM NaCl, 20 mM Tris-HCl, pH 8.0, formamide concentration according to Table S7, 1% blocking reagent, 0.1 g mL^{-1} dextran sulfate, and 0.02% SDS). Humidity chambers were prepared, containing the same formamide concentrations. Samples were hybridized in the humidity chambers for 3 hours at 46°C. Subsequently, samples were washed in washing buffer (20 mM Tris-HCl pH 8.0, 5 mM EDTA pH 8.0, 0.01% SDS, and 0.159 M or 0.08M NaCl for 25% or 35% in the hybridization mixture, respectively) for 15 min at 48°C and afterwards in 1x PBS for 15 min at room temperature. Signal amplification was conducted with 1 µg ml^{-1} A488 tyramids (Thermo Fisher, Wltham, Massachusetts, USA) in amplification buffer (1x PBS, 2 M NaCl, 0.1% blocking reagent, 1 g mL^{-1} dextran sulfate, 0.0015% H_2O_2). Signals were amplified for 45 min at 46°C and samples subsequently washed with deionized water and 96% ethanol. Subsequently, samples were stored overnight at -20°C until further processing.

For direct-geneFISH, samples were hybridized with 62 µg mL^{-1} for each direct-geneFISH probe in hybridization mixture (20 mM EDTA, 5x SSC, 0.1% SDS, 20% dextran sulfate, 0.25 mg ml^{-1} yeast RNA, 0.25 mg ml^{-1} sheared salmon sperm DNA, 1% blocking reagent, 25% formamide; all final concentration). Subsequently, samples were first washed in washing buffer (see above) for 15 min at 48°C, then in 1x PBS for 20 min at room temperature, and last briefly in deionized water. No ethanol washing was conducted, to prevent any potential signal loss. Hybridized filters were counterstained with DAPI as indicated in the main manuscript.

Statistical modelling and visualizations

All statistical analyses done in R (v4.2.2)[14] with the packages brms (v2.19.0)[15], tidyr (v1.3.0)[16], tidybayes (v3.0.4)[17], cmdstanr (v0.5.3)[18], and ez (v4.4-0)[19]. For visualizations we used the packages ggplot (3.4.2)[20], plyr (v1.8.8)[21], ggpubr (v0.6.0)[22], cowplot (v1.1.1)[23], and lubridate (v1.9.2)[24]. The map is retrieved via ggplot from maps (v3.4.1)[25] Color schemes were inspired by the WesAnderson package (v0.3.6)[26].

Supplementary Figures

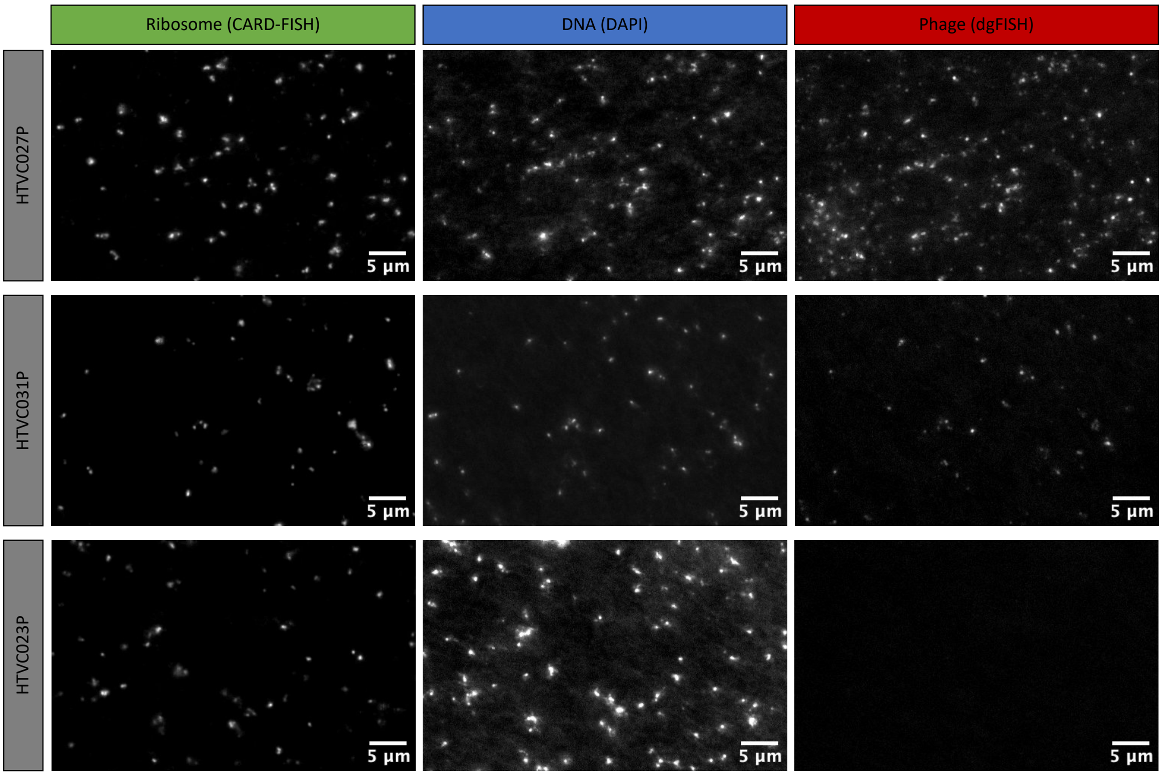

Figure S1 Examples of high-throughput images to determine phage-infected SAR11 cells in control cultures. SAR11 HTCC1062 were infected with HTVC027P, HTVC031P, and HTVC023P. DNA was stained with DAPI, 16S ribosomal RNA with CARD-FISH (SAR11-mix), and phage genes were stained with direct-geneFISH (phage mix for HTVC027P, HTVC031P, and Greip). HTVC023P served as negative control. Images were cropped to a quarter of original size for visualization purposes and scale bars were inserted using Fiji/ImageJ. Example image from n=118 (HTVC023P), n=104 (HTVC027P), and n=92 (HTVC031P) images in total.

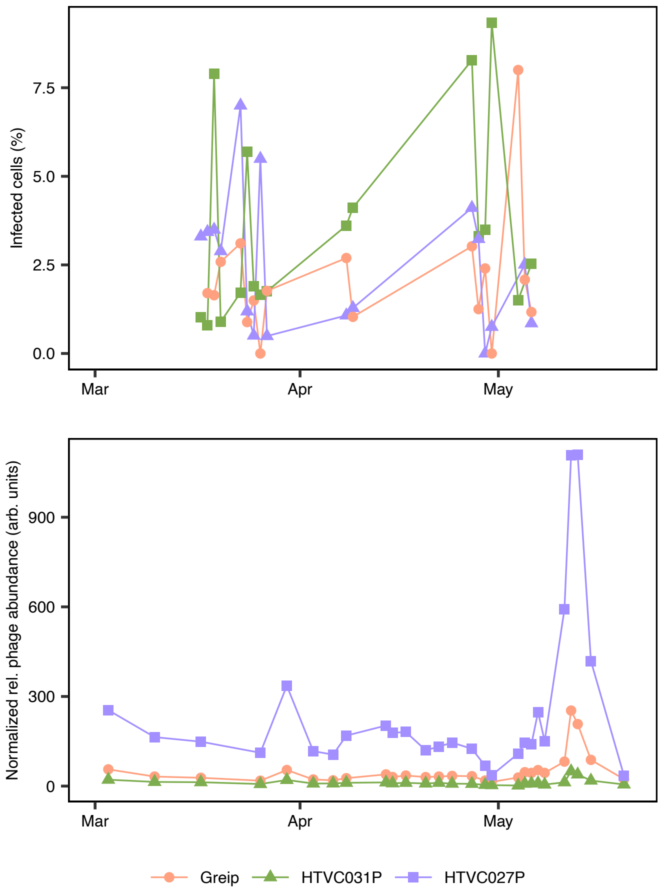

Figure S2 Microscopy-based vs. bioinformatic estimates of phage-infected cells during the 2020 spring phytoplankton bloom at Helgoland Roads. Upper panel: Abundance of phage-infected SAR11 cells per individual phage, based on microscopy estimates. Lower panel: Relative abundances of respective phages were normalized by the total number of reads mapped to all SAR11 MAGs in the same sample.

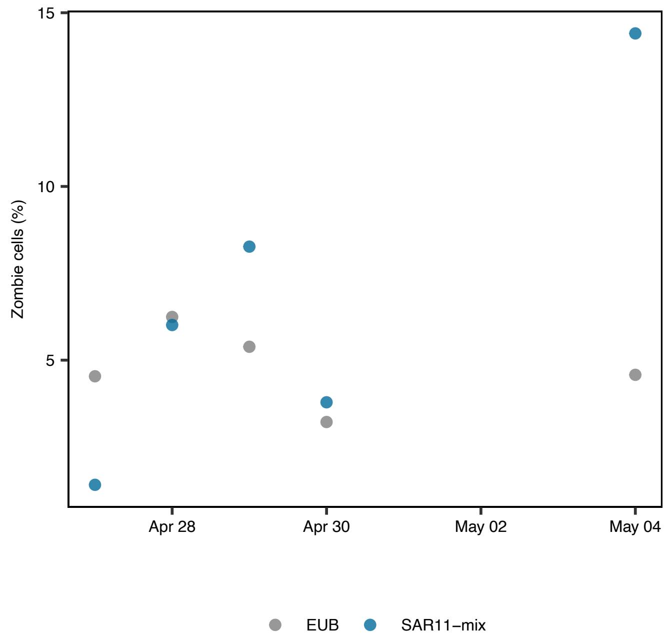

Figure S3 Amount of zombie cells in all bacteria vs. SAR11. Zombie cells as a fraction of total cell counts, determined by DAPI-staining. All bacteria were targeted with the EUB I-III FISH probe, while SAR11 was targeted with the SAR11-mix (table S7).

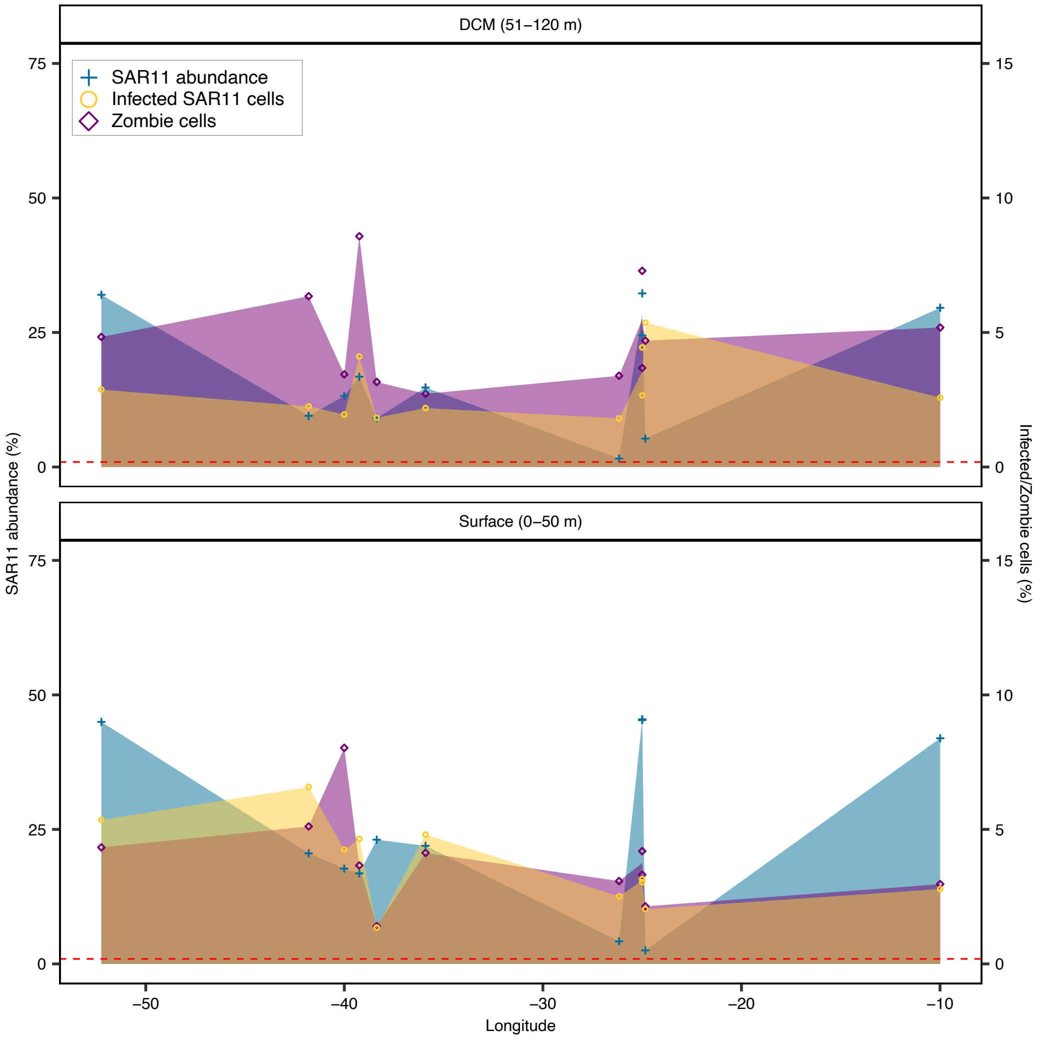

Figure S4 Distribution of SAR11, phage-infected SAR11, and zombie cells in the Southern Ocean. Complete results for Fig. 4.

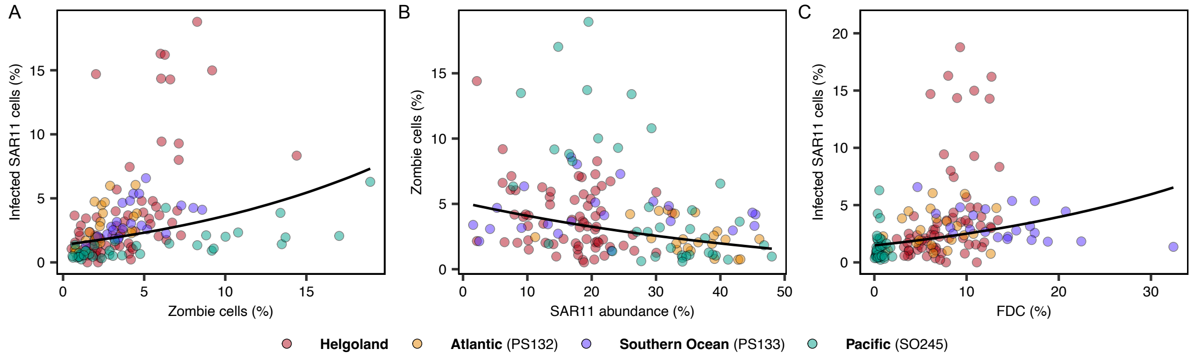

Figure S5 Statistical modelling applying Bayesian beta regression between SAR11 abundance, phage-infected SAR11 cells, and zombie cells. (A) relative abundance of phage-infected SAR11 cells and Zombie cells, (B) relative abundance of Zombie and SAR11 cells, and (C) phage-infected SAR11 cells and frequency of dividing cells (FDC), which is a proxy for cell division activity. Points represent raw data from different sampling campaigns. Line represent data modelled with Bayesian beta regression (back transformed from logit scale).

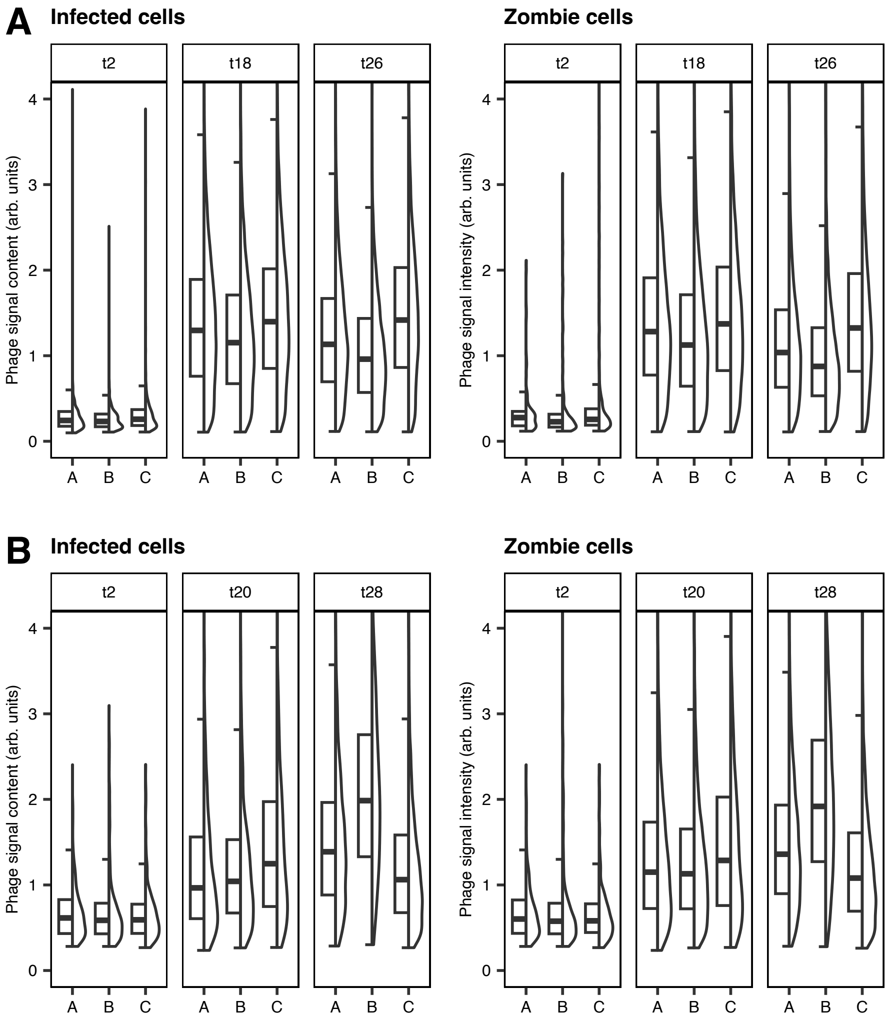

Figure S6 Direct-geneFISH ("phage") signal content from pure cultures. (A) SAR11 infected with HTVC027P. (B) SAR11 infected with HTVC031P. "Infected cells" corresponds to 16S FISH-positive and direct-geneFISH-positive cells, whereas "Zombie cells" do not contain a 16S FISH signal. Boxplots show median and upper and lower quartile of individual cells within each replicate. Whiskers show maximum data points within upper/lower quartile range plus 1.5 times the interquartile range. Outliers not shown. "A", "B", and "C" correspond to replicates.

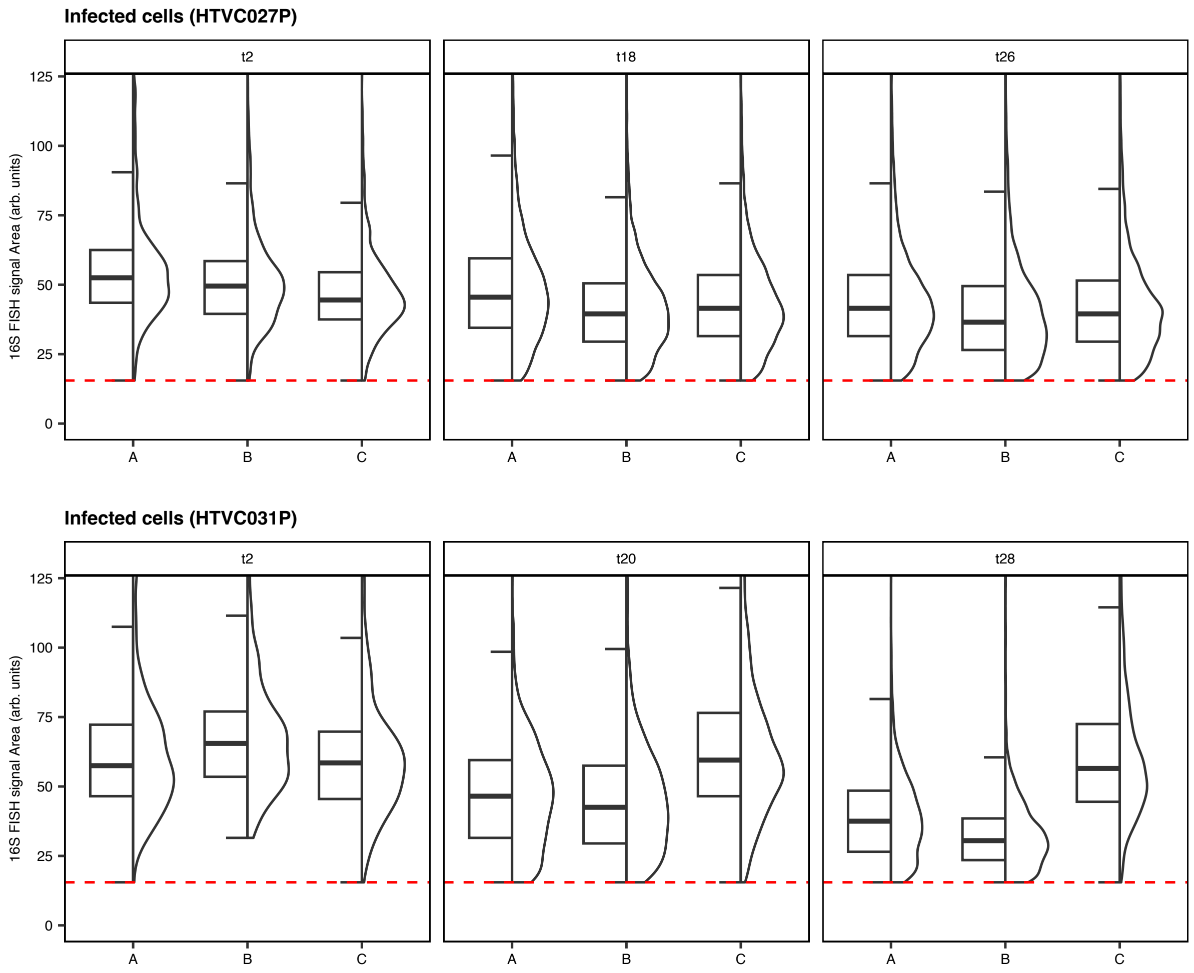

Figure S7 16S FISH fluorescence intensity of phage-infected SAR11 cells from infection experiments. Boxplots show median and upper and lower quartile of individual cells within each replicate. Whiskers show maximum data points within upper/lower quartile range plus 1.5 times the interquartile range. Outliers not shown. Violin plot show data distribution on y-Axis. Red-dashed line represents defined threshold from image analysis. “A”, “B”, and “C” correspond to replicates.

Supplementary Bibliography

1 Sidhu, C. *et al.* Dissolved storage glycans shaped the community composition of abundant bacterioplankton clades during a North Sea spring phytoplankton bloom. *Microbiome* **11**, 1-18 (2023).

2 Zhou, J., Bruns, M. A. & Tiedje, J. M. DNA recovery from soils of diverse composition. *Applied and Environmental Microbiology* **62**, 316-322 (1996).

3 Kolmogorov, M., Yuan, J., Lin, Y. & Pevzner, P. A. Assembly of long, error-prone reads using repeat graphs. *Nature Biotechnology* **37**, 540-546 (2019).

4 Kieft, K., Zhou, Z. & Anantharaman, K. VIBRANT: automated recovery, annotation and curation of microbial viruses, and evaluation of viral community function from genomic sequences. *Microbiome* **8**, 1-23 (2020).

5 O'Leary, N. A. *et al.* Reference sequence (RefSeq) database at NCBI: current status, taxonomic expansion, and functional annotation. *Nucleic acids research* **44**, D733-D745 (2016).

6 Buchholz, H. H. *et al.* Efficient dilution-to-extinction isolation of novel virus–host model systems for fastidious heterotrophic bacteria. *The ISME Journal* **15**, 1585-1598 (2021).

7 Zhang, Z. *et al.* Culturing novel and abundant pelagiphages in the ocean. *Environmental Microbiology* **23**, 1145-1161 (2021).

8 Zhao, Y. *et al.* Pelagiphages in the *Podoviridae* family integrate into host genomes. *Environmental Microbiology* **21**, 1989-2001 (2019).

9 Camacho, C. *et al.* BLAST+: architecture and applications. *BMC Bioinformatics* **10**, 1-9 (2009).

10 Nishimura, Y. *et al.* ViPTree: the viral proteomic tree server. *Bioinformatics* **33**, 2379-2380 (2017).

11 Shaffer, M. *et al.* DRAM for distilling microbial metabolism to automate the curation of microbiome function. *Nucleic acids research* **48**, 8883-8900 (2020).

12 Katoh, K. & Standley, D. M. MAFFT multiple sequence alignment software version 7: improvements in performance and usability. *Molecular Biology and Evolution* **30**, 772-780 (2013).

13 Kearse, M. *et al.* Geneious Basic: an integrated and extendable desktop software platform for the organization and analysis of sequence data. *Bioinformatics* **28**, 1647-1649 (2012).

14 R Core Team, R. R: A language and environment for statistical computing. (2022).

15 Bürkner, P.-C. Advanced Bayesian multilevel modeling with the R package brms. *arXiv preprint arXiv:1705.11123* (2017).

16 Wickham, H., Vaughan, D. & Girlich, M. tidyr: Tidy Messy Data. *tidyr: Tidy Messy Data* (2023).

17 Kay, M. tidybayes: Tidy data and geoms for Bayesian models. *R package version* **2**, 1 (2023).

18 Gabry, J. & Češnovar, R. cmdstanr: R Interface to'CmdStan'. *URL: https://mc-stan. org/cmdstanr, https://discourse. mc-stan. org* (2022).

19 Lawrence, M. A. & Lawrence, M. M. A. Package 'ez'. *R package version* **4** (2016).

20 Wickham, H. *ggplot2*. Vol. 3 (Springer-Verlag New York, 2011).

21 Wickham, H. The split-apply-combine strategy for data analysis. *Journal of statistical software* **40**, 1-29 (2011).

22 Kassambara, A. & Kassambara, M. A. ggpubr: 'ggplot2' Based Publication Ready Plots. (2020).

23 Wilke, C. cowplot: streamlined plot theme and plot annotations for 'ggplot2'. R package version 1.1. 1. (2020).

24 Grolemund, G. & Wickham, H. Dates and times made easy with lubridate. *Journal of statistical software* **40**, 1-25 (2011).

25 Becker, R. A., Wilks, A. R., Brownrigg, R., Minka, T. P. & Deckmyn, A. maps: Draw geographical maps. *R package version* **3.4.1** (2022).

26 Ram, K. & Wickham, H. wesanderson: A Wes Anderson palette generator. *R package version 0.3* **6** (2018).
