# Supplementary material for: Globally occurring pelagiphage infections create ribosome-deprived cells
Source: Nat Commun. 2024 May 2;15:3715. doi: 10.1038/s41467-024-48172-w (PMC11066056; doi:10.1038/s41467-024-48172-w)
Supplement: Supplementary file 3 — Description of Additional Supplementary Files [file 41467_2024_48172_MOESM3_ESM.pdf]

Description of Additional Supplementary Files

File Name: Supplementary Data 1
Description: Proportion of cells - cell counts as 16S FISH positive, phage-infected, and Zombie cells in control cultures. Note that HTVC023P is the negative control.

File Name: Supplementary Data 2
Description: ACME tool settings for automated image analysis.

File Name: Supplementary Data 3
Description: Identification of zombie cells specific to SAR11 and all bacteria. The same phage-probe mix was used for both experiments.

File Name: Supplementary Data 4
Description: Anti-phage defense systems in Pelagibacter ubique and SAR11 metagenomic assembled genomes, identified with DefencseFinder.

File Name: Supplementary Data 5
Description: DRAM annotation output of phage reference genomes.

File Name: Supplementary Data 6
Description: Metadata and microscopy raw data for cruise samplings.

File Name: Supplementary Data 7
Description: FISH probes used in this study with corresponding labels and formamide concentrations.
