# Supplementary material for: Globally occurring pelagiphage infections create ribosome-deprived cells
Source: Nat Commun. 2024 May 2;15:3715. doi: 10.1038/s41467-024-48172-w (PMC11066056; doi:10.1038/s41467-024-48172-w)
Supplement: Supplementary file 5 — Reporting Summary [file 41467_2024_48172_MOESM5_ESM.pdf]

nature portfolio

Corresponding author(s): Jan D. Brüwer
Bernhard M. Fuchs

Last updated by author(s): Apr 4, 2024

Reporting Summary

Nature Portfolio wishes to improve the reproducibility of the work that we publish. This form provides structure for consistency and transparency in reporting. For further information on Nature Portfolio policies, see our Editorial Policies and the Editorial Policy Checklist.

Statistics

For all statistical analyses, confirm that the following items are present in the figure legend, table legend, main text, or Methods section.

n/a	Confirmed	
☐	☒	The exact sample size (*n*) for each experimental group/condition, given as a discrete number and unit of measurement
☐	☒	A statement on whether measurements were taken from distinct samples or whether the same sample was measured repeatedly
☐	☒	The statistical test(s) used AND whether they are one- or two-sided<br>*Only common tests should be described solely by name; describe more complex techniques in the Methods section.*
☐	☒	A description of all covariates tested
☐	☒	A description of any assumptions or corrections, such as tests of normality and adjustment for multiple comparisons
☐	☒	A full description of the statistical parameters including central tendency (e.g. means) or other basic estimates (e.g. regression coefficient) AND variation (e.g. standard deviation) or associated estimates of uncertainty (e.g. confidence intervals)
☐	☒	For null hypothesis testing, the test statistic (e.g. F, t, r) with confidence intervals, effect sizes, degrees of freedom and P value noted<br>*Give P values as exact values whenever suitable.*
☐	☒	For Bayesian analysis, information on the choice of priors and Markov chain Monte Carlo settings
☒	☐	For hierarchical and complex designs, identification of the appropriate level for tests and full reporting of outcomes
☒	☐	Estimates of effect sizes (e.g. Cohen's *d*, Pearson's *r*), indicating how they were calculated

Our web collection on statistics for biologists contains articles on many of the points above.

Software and code

Policy information about availability of computer code

Data collection	AxioVision software with custom-developed MPISYS plugin. Automated Cell Measuring and Enumeration tool (ACME; freely available at https://www.mpi-bremen.de/automated-microscopy.html).
Data analysis	VIBRANT (v1.2.0), VipTree (v1.1.2), DRAM-v, Geneious (v2022.1.1) with MAFFT (v7.450) plugin;<br>ACME Tool (v3) available from https://www.mpi-bremen.de/automated-microscopy.html#section19794 ;<br>Fiji/ImageJ with the MicrobeJ (v5.12.d) plug-in;<br>R (v4.2.2) with packages brms (v2.19.0), tidyr (v1.3.0), tidybayes (v3.0.4), cmdstanr (v0.5.3), ez (v4.4-0), ggplot (3.4.2)20, plyr (v1.8.8)21, ggpubr (v0.6.0), cowplot (v1.1.1), lubridate (v1.9.2), maps (v3.4.1). Color schemes were inspired by the WesAnderson package (v0.3.6).<br><br>All scripts are available at: https://gitlab.mpi-bremen.de/jbruewer/pelagiphage-abundance

For manuscripts utilizing custom algorithms or software that are central to the research but not yet described in published literature, software must be made available to editors and reviewers. We strongly encourage code deposition in a community repository (e.g. GitHub). See the Nature Portfolio guidelines for submitting code & software for further information.

Data

Policy information about availability of data

All manuscripts must include a data availability statement. This statement should provide the following information, where applicable:

- Accession codes, unique identifiers, or web links for publicly available datasets
- A description of any restrictions on data availability
- For clinical datasets or third party data, please ensure that the statement adheres to our policy

The microscopy data generated in this study have been deposited in the Edmond database [https://doi.org/10.17617/3.3ZLOAT]. Metagenomes assessed in this study are publicly available from the European Nucleotide Archive (ENA) with the accession code PRJEB52999 [https://www.ebi.ac.uk/ena/browser/view/PRJEB52999].
In this manuscript, the following databases were used: GTDB release 202 [https://data.gtdb.ecogenomic.org/], RefSeq (online via NCBI website) as of October 22nd 2023 [ftp://ftp.ncbi.nlm.nih.gov/refseq/release/viral], and the nt database of the NCBI blast server as of February 14th 2023.

Research involving human participants, their data, or biological material

Policy information about studies with human participants or human data. See also policy information about sex, gender (identity/presentation), and sexual orientation and race, ethnicity and racism.

Reporting on sex and gender	nA
Reporting on race, ethnicity, or other socially relevant groupings	nA
Population characteristics	nA
Recruitment	nA
Ethics oversight	nA

Note that full information on the approval of the study protocol must also be provided in the manuscript.

Field-specific reporting

Please select the one below that is the best fit for your research. If you are not sure, read the appropriate sections before making your selection.

☐ Life sciences ☐ Behavioural & social sciences ☒ Ecological, evolutionary & environmental sciences

For a reference copy of the document with all sections, see nature.com/documents/nr-reporting-summary-flat.pdf

Ecological, evolutionary & environmental sciences study design

All studies must disclose on these points even when the disclosure is negative.

Study description	We quantified phage-infected cells, using high-throughput imaging and image analysis of environmental samples. To quantify phage-infected cells, we used a nested approach: A cell required a DNA in order to be quantified as phage-infected cell. Phage-infected SAR11 cells, additionally needed a 16S rRNA FISH signal. Quantification was done automatically and based on recorded images. We analyzed a time-series data with replication over time (67 data points), as well as three oceanic transects with 11 (Atlantic and Southern Ocean) and 15 (Pacific) stations.
Research sample	Bacterioplankton samples were fixed with 1% formaldehyde for one hour at room temperature and subsequently immobilized on polycarbonate filters with 0.2 µm pore size. For environmental samples, water was collected within a Niskin bottle, attached to a CTD rosette, or through an in situ pump.<br>SAR11 abundances, cell division rate and chlorophyll a values for the Helgoland dataset have been retrieved from Brüwer et al. 2023, mSystems, and SAR11 abundances from Pacific samples have been retrieved from Reintjes et al. 2017, AEM. We addressed this appropriately in the manuscript.
Sampling strategy	No a priori sample-size calculations were performed. During the time-series, samples were taken on each working day, unless weather conditions did not allow a sampling (67 samples over 133 days). During cruises, samples were collected at every station (11 for Atlantic and Southern Ocean, 15 for pacific), which is limited due to ship-time.
Data collection	After processing of samples in the wet lab (i.e., fluorescence in situ hybridization), filter samples were embedded on microscopy slides. Samples were imaged on an automatic microscope with fixed settings, including a fixed exposure time. Cell abundance and phage-infected cells were determined using high-throughput image analysis to prevent a human bias. All data processing was done by first author J.D. Brüwer.

Timing and spatial scale	Sampling times were as follows: Helgoland (54° 11.3'N, 7° 54.0' E) 2nd March to 13th July 2020; Atlantic (North - South transect along the African coast; 47.9°N-29.5°S, 8.6°W-12.3°E) 3rd September 2022 to 27th September 2022; Southern Ocean (East-West Transect with focus on Sandwich Island; 49.1°-55.8°S, 10.9-24.8°W) 1st October to 17th November 2022; Pacific (East-West Transect from Chile to New Zealand through South Pacific Gyre; 23.5°-39°S, 84.6-170.0°W) 25th January 2015 to 24th January 2016. All samples were processed and data collected between December 2022 and March 2023.
Data exclusions	No data was excluded from the analysis.
Reproducibility	For each experimental approach, positive control (i.e., phages from a pure culture) and negative controls were applied. Controls were visually inspected manually and were successful every time.<br>All recorded images are available online, ensuring reproducibility of our high-throughput image analysis.
Randomization	Samples were grouped by expedition. Experiments were controlled by simultaneous sampling of a positive and negative control. Within each group, samples were randomized for laboratory processing.
Blinding	Images and image analysis were conducted automatically with global settings, hence no blinding was necessary.

Did the study involve field work? ☒ Yes ☐ No

Field work, collection and transport

Field conditions	Time-series from Helgoland: Sampling during a phytoplankton spring bloom (chlorophyll a ranging from 0.7 to 9.4 µg /L; water temperature increased from 7°C to 13°C).<br><br>Global sampling was conducted during three cruises. Sampled water depths ranged from 0 to 150 m (see table S6 for details). Sea surface temperatures were in the ranges of 17 to 25°C (Pacific), 16 to 25°C (Atlantic), and 1 to 18°C (Southern Ocean cruise)
Location	Helgoland: Long-term ecological research station Kabeltonne; 54° 11.3'N, 7° 54.0' E; surface water (~1 m depth)<br>Atlantic: North-South transect along the east-coast of Africa; 47.9°N-29.5°S, 8.6°W-12.3°E; 10-100 m depth<br>Southern Ocean: East-West Transect with focus close to Sandwich islands; 49.1°-55.8°S, 10.9-24.8°W; 20 -150 m depth<br>Pacific: East-West Transect from Chilean upwelling through the oligotrophic gyre; 23.5°-39°S, 84.6-170.0°W; 20-100 m depth<br><br>Please note: Exact sampling locations and depths are specified in Table S2.
Access & import/export	Helgoland: Collaboration with Alfred Wegener Instiute, Germany, who maintain the long-term ecological research station Kabeltonne. No research permission required.<br>Atlantic: Cruise with R/V Polarstern (Alfred Wegener Institute, Bremerhaven); Sampling beyond EEZ. No permission required<br>Southern Ocean: Cruise with R/V Polarstern (Alfred Wegener Institute, Bremerhaven, Germany); Nagoya agreement ongoing with Argentina<br>Pacific: Cruise with R/V Sonne (University Hamburg, Hamburg, Germany); Sampling beyond EEZ. No permission required.<br><br>Analyzed samples do not fall under the regulations of the Nagoya protocol.
Disturbance	No disturbances were caused.

Reporting for specific materials, systems and methods

We require information from authors about some types of materials, experimental systems and methods used in many studies. Here, indicate whether each material, system or method listed is relevant to your study. If you are not sure if a list item applies to your research, read the appropriate section before selecting a response.

Materials & experimental systems

n/a	Involved in the study
☒	☐ Antibodies
☒	☐ Eukaryotic cell lines
☒	☐ Palaeontology and archaeology
☒	☐ Animals and other organisms
☒	☐ Clinical data
☒	☐ Dual use research of concern
☒	☐ Plants

Methods

n/a	Involved in the study
☒	☐ ChIP-seq
☒	☐ Flow cytometry
☒	☐ MRI-based neuroimaging
